# Validation of the Geriatric Depression Scale for an elderly Sri Lankan clinic population

**DOI:** 10.4103/0019-5545.70979

**Published:** 2010

**Authors:** M Kulathunga, S Umayal, S Somaratne, S Srikanth, S Kathriarachchi, KRD De Silva

**Affiliations:** Department of Psychiatry, Sri Lanka; 1 Genetic Diagnostic & Research Laboratory and Human Brain Tissue Repository, Department of Anatomy, Faculty of Medical Sciences, University of Sri Jayewardenepura, Nugegoda, Sri Lanka; 2 Department of Botany, Open University of Sri Lanka, Sri Lanka; 3 Neurology unit, Apollo Hospital, Colombo - 05, Sri Lanka

**Keywords:** GDS, depression, Sri Lanka

## Abstract

**Background:**

Geriatric Depression Scale (GDS) has not been validated for the elderly population in Sri Lanka.

**Aim:**

To translate, validate, and examine the effectiveness of GDS and to suggest the optimal cut-off scores for elderly Sri Lankans attending a psychogeriatric clinic.

**Materials and Methods:**

The Sinhalese translation of GDS (GDS-S) was administered to people aged 55 years and above, attending a psychogeriatric outpatient clinic. The diagnostic performance of the instrument was compared against the ICD 10 diagnosis of a consultant psychiatrist, which was considered the ‘gold standard’. Receiver operating characteristic (ROC) analysis was carried out to compare the diagnostic performance of the GDS-S. Optimal cut-off scores for depression and sensitivity and the specificity of the instrument was determined.

**Results:**

A total of 60 subjects formed the final sample (male/female=16/44) of which 30 were depressed, while 30 were age- and sex-matched controls. The optimal cut-off score for GDS-S was 8 for differentiating non-depressed from mildly depressed, while the cut-off score for moderate depression was 10. Sensitivity and specificity of GDS-S was 73.3% for differentiating depressed from non-depressed.

**Conclusion:**

GDS is culturally acceptable, easy to use, sensitive, and a valid instrument to diagnose depression and to differentiate mild from moderate depression in an elderly Sri Lankan clinic population.

## INTRODUCTION

In the last decade the elderly population living in developing countries has increased by 200–280% compared with a mere 30–40% increase in the developed nations.[1] In 2002, the percentage of the population aged over 60 years, in Sri Lanka, was 10%, but it is estimated to reach 28% by 2050–this is the highest estimated value for South – Central Asia and the second highest for South East Asia.[2] Depression is one of the most common psychiatric disorders affecting the elderly and is a major public health problem.[3]

Lack of valid diagnostic instruments specific to the local settings is one of the main reasons behind poor assessment of depression. This is moreso in the elderly, as they often tend not to seek help for psychological problems and instead present to physicians, focusing on somatic symptoms. The best means of overcoming the under recognition is routine screening, using an instrument that is easy to administer and highly effective.[4,5]

Geriatric Depression Scale (GDS) is one of the most widely used scales for depression detection.[6] The original GDS was developed by Yesavage *et al*.[7] Depression scales are used commonly in Western countries and in a few developing countries: For example, India,[8] Singapore,[9] and Korea.[10] Although many depression scales exist, they have not been validated for the elderly population in Sri Lanka.

### Aim

The aim of this study is to translate, validate, and examine the effectiveness of GDS and to suggest the optimal cut-off scores for the elderly Sri Lankans attending a psychogeriatric clinic.

## MATERIALS AND METHODS

Thirty patients, over 55 years of age, diagnosed as having depressive disorder, attending a psycho-geriatric outpatient clinic at the Colombo South Teaching Hospital, Kalubowila, and 30 age-and sex-matched controls accompanying patients to the same clinic were administered the translated GDS Sinhalese version (GDS-S). All included in the study had no other concomitant psychiatric illnesses and were able to communicate. Ethical clearance was obtained from the Faculty of Medical Sciences, University of Sri Jayewardenepura. Informed written consent was obtained from all participants.

### Procedure

The 15-item GDS needed no modification and was translated directly to Sinhalese language and the score ranged between 0 and 15. The translated scale was back translated to English by a different group and the modified instrument was pilot tested on a random sample of 30 elderly people, prior to use in the present study.

All subjects were assessed by a consultant psychiatrist with a special interest in psychogeriatrics, who diagnosed depression according to the International Classification of Diseases (ICD-10).[11] Age- and sex-matched controls were selected by an independent doctor conducting the clinic. Finally, the depression scale was administered to each subject by a research assistant, blind to the subject’s clinical status. The diagnostic performance of the instrument was then compared against the clinical diagnosis, which was considered the ‘gold standard’.

### Data Analysis

All analyses were carried out using the SPSS Version 13.0 Software (2005, Chicago). Statistical significance was set at *P*<0.05. Receiver operating characteristic (ROC) analysis was carried out to assess the diagnostic performance of the GDS-S. The optimal cut-off scores for depression and the sensitivity and specificity were determined.

## RESULTS

### Demographics

The final cohort consisted of 30 depressed elderly subjects and 30 controls. The age and sex distributions of the study sample are shown in Table 1. There was a female preponderance (*n*=44, 73.3%) in the sample. The majority of the subjects (*n*=30, 50%) were between 55 and 64 years of age, and the age difference between the depressed and non-depressed groups was not statistically significant (*P*>0.05), while the difference in the gender proportions between the two groups was statistically significant (*P*<0.05).

**Table 1:** Demographic characteristics

	Non-depressed (n=30) n (%)	Depressed (n =3 0) n (%)	Total (n=60) n (%)
**Age (years)**			
55–64	16 (53.3)	14 (46.6)	30 (50)
65–74	13 (43.3)	14 (46.6)	27 (45)
≥75	01 (3.3)	02 (6.6)	03 (5)
**Gender**			
Male	10 (33.3)	06 (20)	16 (26.7)
Female	20 (66.6)	24 (80)	44 (73.3)
Median age	64 (55–80)	65 (56–77)	64.5 (55–80)

n=total number of subjects

The mean age for depressed and non-depressed females was 63.83 and 65.05, respectively, while the mean age for depressed and non-depressed males was 66.16 and 62.5, respectively.

### Diagnostic performance of the instrument

The optimal cut-off score for GDS-S was 8 in differentiating non-depressed from mildly depressed, while the cut off score for moderate-to-severe depression was 10. Both sensitivity and specificity of the GDS-S was 73.3%. Area under the curve was 0.436 (95% CI 0.288-0.436) [Table 2].

**Table 2:** Descriptive scores and results of the ROC analysis of GDS -S

	Total n=60	Mild depression n=9	Moderate depression n=21
**GDS-S scores**			
Mean (Std. Err)	6.27 (0.467)	5.33 (0.601)	10.52 (0.429)
Median (range)	5.5 (2–14)	6 (2–8)	10(8–14)
Optimal cut-off score	8	8	10
Sensitivity	73.3%	77.8%	85.7%
Specificity	73.3%	72.5%	84.6%
Area under ROC	0.436	0.617	0.505
(95% CI)	(0.288–0.583)	(0.378–0.855)	(0.352–0.659)

P<0.05

‘Feel life is empty’ and ‘feeling hopeless about the future’ were the best items for recognizing depression using GDS-S.

## DISCUSSION

Diagnosing depressive disorder in the elderly needs more emphasis in developing countries. In this respect, the validating instrument used in local settings is of paramount importance.

The optimal cut-off score of our study for GDS-S is higher when compared with cut-off scores from other studies. In Lim *et al*.’s[9] study on diagnosing depression among the cognitively intact elderly Chinese in Singapore, the optimal cut-off score is three-fourth (sensitivity 84.0%, specificity 85.7%).[9] These cut-off scores are much lower than the score of 8 obtained in our study (sensitivity 73.3%, specificity 73.3%). On the other hand, Cwikel and Ritchie (1989),[12] using a sample of 71 Israeli elderly subjects who had been suspected of suffering from depression, reported a higher GDS optimal cut-off score of 7/8 (sensitivity 72%, specificity 75%), which is consonant to the findings of our study. However, validation of the translated, shortened GDS has also been done in the Dutch elderly population,[13] which proposed an optimal cut off score of 2/3 (sensitivity 76%, specificity 53%). The different cut-off scores and varying sensitivities and specificities of shortened GDS highlights, among other matters, the need to consider cross-cultural differences when screening for depression.

The GDS primarily taps the affective and motivational cognitive components of depression in the elderly. It deliberately forgoes examining the neuro-vegetative and somatic components that could result from a variety of non-psychiatric medical conditions in older persons and thus create false positive responses.[5] Thus, it excludes symptoms such as disturbance in sleep, appetite, and weight even though these symptoms are essential for the diagnosis of major depression.[5]

Ganguli and Gupta measured depressive symptomatology in a largely illiterate elderly population in India, using a new Hindi version of the Geriatric Depression Scale (GDS-H), to examine its distribution, as also its association with age, gender, literacy, cognitive impairment, and functional impairment. Greater numbers of depressive symptoms were measured by higher scores on the GDS-H, which was associated with older age and illiteracy. Among the illiterate, there was no gender difference, while among the literate, higher GDS-H scores were found among women. In conclusion Ganguli found that depressive symptoms, as measured by the GDS-H, were predominant in this elderly illiterate north Indian population and was strongly associated with both cognitive and functional impairment.[8]

Shah *et al* stated that an adequate screening questionnaire for depression should have sensitivity and specificity of at least over 70%,[14] and from that perspective, the Sinhalese version of GDS (sensitivity 73.3%, specificity 73.3%) can certainly be considered a valid screening instrument and can also be used in the general population.

In conclusion the GDS-S was found to be an easy to administer and culturally acceptable instrument to diagnose depression in outpatient settings in Sri Lanka. It was effective in differentiating non-depressed from depressed and mildly depressed from those who were moderately depressed.

## References

[1] Help age India. Aging scenario. http://www.helpageindia.com.

[2] (2002). United Nations. Population ageing. Population Division, Department of Economic and Social Affairs.

[3] Murray CJ, Lopez AD (1997). Alternative projections of mortality and disability by cause 1990-2020: Global Burden of Disease Study. Lancet.

[4] Brind TL, Yesavage JA, Owen L (1982). Screening tests for geriatric depression. Clin Gerontol.

[5] Sheikh JI, Yesavage JA, Brooks JO, Friedman L, Gratzinger P, Hill RD (1991). Proposed factor structure of the Geriatric Depression Scale. Int Psychogeriatr.

[6] Montorio I, Izal M (1996). The Geriatric Depression Scale: A review of its development and utility. Int Psychogeriatr.

[7] Yesavage JA, Brink TL, Rose TL, Lum O, Huang V, Day M (1983). Development and validation of a geriatric depression screening scale: A preliminary report. J Psychiatry Res.

[8] Ganguli M, Dude S, Jhonson JM, Pandav R, Chandra V, Dodge HH (1999). Depressive symptoms, cognitive impairment and functional impairment in a rural elderly population in India: A Hindi version of Geriatric Depression Scale (GDS-H). Int J Geriatr Psychiatry.

[9] Lim PP, Ng LL, Chiam PC, Ong PS, Ngui FT, Sahadevan S (2000). Validation and comparison of three brief depression scales in an elderly Chinese population. Int J Geriatr Psychiatry.

[10] Han C, Jo SA, Kwak JH, Pae CU, Steffens D, Jo I (2008). Validation of the Patient Health Questionnaire - 9 Korean version in the elderly population: The Ansan Geriatric study. Compr Psychiatry.

[11] (1992). World Health Organization, International Classification of Diseases, 10th revision.

[12] Cwikel J, Ritchie K (1989). Screening for depression among the elderly in Israel: An assessment of the short Geriatric Depression Scale (S-GDS). Isr J Med Sci.

[13] Marwijk HWJV, Wallace P, DeBrock GH, Hermans J, Kaptein AA, Mulder JD (1995). Evaluation of the feasibility, reliability and diagnostic value of shortened version of the Geriatric Depression Scale. Br J Gen Pract.

[14] Shah A, Phongsathorn V, George C, Bielawska C, Katona C (1992). Psychiatric morbidity among continuing care geriatric inpatients. Int J Geriatr Psychiatry.

